# Guessing Meaning From Word Sounds of Unfamiliar Languages: A Cross-Cultural Sound Symbolism Study

**DOI:** 10.3389/fpsyg.2019.00593

**Published:** 2019-03-19

**Authors:** Anita D’Anselmo, Giulia Prete, Przemysław Zdybek, Luca Tommasi, Alfredo Brancucci

**Affiliations:** ^1^Department of Psychological, Health and Territorial Sciences, ‘G. d’Annunzio’ University of Chieti-Pescara, Chieti, Italy; ^2^Institute of Psychology, University of Opole, Opole, Poland

**Keywords:** sound symbolism, natural languages, psycholinguistic, iconicity, cross-linguistic

## Abstract

Sound symbolism refers to a non-arbitrary relationship between the sound of a word and its meaning. With the aim to better investigate this relationship by using natural languages, in the present cross-linguistic study 215 Italian and Polish participants were asked to listen to words pronounced in 4 unknown non-indo-European languages (Finnish, Japanese, Swahili, Tamil) and to try to guess the correct meaning of each word, by choosing among 3 alternatives visualized on a computer screen. The alternatives were presented in the mother tongue of participants. Three different word categories were presented: nouns, verbs and adjectives. A first overall analysis confirmed a semantic role of sound symbols, the performance of participants being higher than expected by chance. When analyzed separately for each language and for each word category, the results were significant for Finnish and Japanese, whereas the recognition rate was not significantly better than chance for Swahili and Tamil. Results were significant for nouns and verbs, but not for adjectives. We confirm the existence of sound symbolic processing in natural unknown languages, and we speculate that some possible difference in the iconicity of the languages could be the basis for the difference we found. Importantly, the evidence that there were no differences between Italian and Polish participants allows us to conclude that the sound symbolism is independent of the mother tongue of the listener.

## Introduction

A central assumption of modern linguistics is that the relationship between the acoustic features of a word and its meaning is fundamentally arbitrary. The notion formulated by de Saussure about arbitrariness argues that a systematic connection does not exist between the sound of a word and its semantic concept (De Saussure, 1916; Hockett, 1977). Furthermore, arbitrariness is considered a distinctive feature of spoken language that differentiates it from other communication systems. Although this seminal assumption regarding the arbitrariness of natural language has been widely acknowledged for decades, a growing body of literature suggests that non-arbitrary correspondences between sound structure and linguistic category also exist, and that listeners with unrelated language experiences can be sensitive to these correspondences (Sapir, 1929; Ramachandran and Hubbard, 2001; Nygaard et al., 2009; Kovic et al., 2010).

A long history of sound symbolism research has investigated the existence of a correspondence between speech sounds and semantic dimensions. Most of the first studies focused on the exploration of a relation between syllables and the size or shape of visual stimuli. Such relations have often been observed with non-linguistic words and have been defined *synesthetic* sound symbolism (Hinton et al., 2006). For instance, Sapir (1929) presented two trigram non-words varying only for the vowel “a” or “i” (e.g., ”*Mal*”-”*Mil*”), and asked participants to associate each non-word with either a larger or a smaller object. He found that participants preferred the word Mal rather than Mil, containing the vowel sound “a”, to refer to the larger object (see also Newman, 1933). In the same years Köhler (1929), in the Book “Gestalt Psychology”, and more recently Ramachandran and Hubbard (2001); Westbury (2005) and Maurer et al. (2006), reported that when subjects were presented with a curvy round shape and a spiky angular shape, most of them matched the curvy shape with the nonsense word “*maluma*” or “*bouba*” and the spiky shape with the nonsense word “*takete*” or “*kiki*”. This occurred even though they had never seen these stimuli before. These results indicate that there is a crossmodal correspondence between sound structure of a word and perceptual properties of the figures (shapes), in a process that occurs implicitly and automatically.

These findings led to a growing stream of research in the topic of sound symbolism also with natural languages, demonstrating that a relation between the sound structure of a word and the lexical class or meaning may also exist. Furthermore, the idea that sound symbolism is universal and could be applied to many languages has also been validated by means of studies based on a cross-linguistic approach. For instance, it has been shown that people can correctly match antonym pairs, with the correct meaning in an unfamiliar language: Tsuru and Fries, 1933, developed one of the first experiments in this domain, finding that English speaking participants matched 69% of Japanese antonym pairs to their correct English translations (e.g., “*hayai-osoi”* meaning “*fast*-*slow”*). This paradigm was replicated in a study by Brown et al. (1955), in which native English participants were presented with antonyms (e.g., warm-cool, heavy-light, bright-dark) indicating sensory experiences, in unfamiliar languages (Chinese, Czechia, and Hindi). Participants performed the task better than chance using only the sounds of the words to guess the words meaning (see also Klank et al., 1971; Kunihira, 1971; Berlin, 2006). After this, similar studies have been performed introducing some variations in the original paradigm of Brown et al. (1955). These studies extended stimulus material previously restricted to word pairs expressing antonymic concepts. They demonstrated that subjects performed the tasks better than chance although the accuracy rate was sensitive to changes in the experimental procedure (Maltzman et al., 1956; Brackbill and Little, 1957; Brown and Nuttall, 1959). More recent studies have been performed extending these results to multiple languages (Tzeng et al., 2017), furthermore the existence of a cross-modal correspondence between sound word and meaning has been supported by a study in synesthesia. This study found a stronger sensibility to sound symbolism in synesthetes compared to normal population, as a result of an exaggeration of their general cross-modal associations (Bankieris and Simner, 2015).

The correspondence between word sound and meaning suggests the existence of a non-arbitrary relation between them and offers an advantage in language processing possibly facilitating e.g., word learning (Perniss et al., 2010; Lockwood et al., 2016). In a recent research, for example, Imai et al. (2008) using an implicit verb learning task, demonstrated that Japanese adults, 2-year-old Japanese children, and even English speakers with no knowledge of Japanese, were sensitive to sound symbolic relations between novel sounds, verbs, and actions. These results have been confirmed also in experiments with English children, providing evidence of cross-linguistic early sensitivity toward sound symbolism (Kantartzis et al., 2011; Yoshida, 2012). Nygaard et al. (2009), in an explicit word learning experiment, found that English speaking participants presented with Japanese-English antonyms, were faster in learning and responding to Japanese words matched with the correct English translation, than Japanese words matched with the opposite or unrelated English translation.

Starting from the idea of a non-arbitrariness of the acoustic form of the words and considering previous promising results in the domain of sound symbolism, the aim of the present study was to shed more light on the way in which iconicity, namely the similarity between sign and referent, determines a non-arbitrary link between sound and meaning, leading to a correct recognition of new words. Specifically, we carried out an experiment on phonetic symbolism, taking into account natural languages. In this study we tested two groups of participants: a group of native Italian speakers and a group of native Polish speakers, who were presented with vocabulary items in languages unknown to them, that is: Finnish, Japanese, Tamil, and Swahili. All these 4 languages do not belong to the Indo-European language family and for this reason they should have weak historical relationships and resemblances with Italian and Polish, the mother tongues of participants. As regards Finnish, although it is a language spoken in a European country, such as Italian and Polish, it does not share the same linguistic origin of these two languages; Finnish in fact belongs to the group of Uralic languages, whereas both Italian and Polish belong to the Indo-European group. The second language presented is Japanese, spoken primarily in Japan and belonging to the Japonic language family. As regards Swahili, the third language, it belongs to the Atlantic-Congo family, spoken in the east coast of Africa. Finally, Tamil is a Dravidian language predominantly spoken in India and Sri Lanka (Hammarström et al., 2017).

We hypothesized that although participants were presented with languages they did not know and with which they were unfamiliar, they could guess the correct translations of words better than chance because sound symbolic cues would implicitly introduce perceptual analogies between the acoustic form and the meaning of the words, facilitating their recognition. For this reason, we presented open-class words: verbs, nouns and adjectives, that are classified as content words as they convey semantic information (Furtner et al., 2011). These categories of words were chosen as they could be more iconic and more related to concrete representation when compared with closed-class words (pronouns, conjunctions, etc.; Furtner et al., 2009). Furthermore, we wanted to investigate if the relationship between perceptual form and meaning is variable among verbs, nouns, and adjectives. We expected that the sound symbolic relation could lead to a different performance among categories. For example it has been suggested that adjectives depicting sensory and motor experience are more likely to be iconic, and this could facilitate the recognition of meaning, but we do not exclude that there may be differences among the languages used in the present study (Perry et al., 2015). Furthermore we examined sound symbolism with languages from different rhythmic classes. The rhythmic properties of a language are an important aspect in speech processing, so that it is one of the first attributes acquired in the native tongue. Infants, for example, show an early knowledge of the sound organization of their native language (Nazzi et al., 2000). In this view, we presented two syllable-timed languages (Finnish and Swahili) akin to mother tongues of participants (Italian and Polish), and two mora-timed languages (Japanese and Tamil). Given the importance of rhythmic structure of the speech in conveying information crucial for vocal communication (Tzeng et al., 2017), we took into consideration that a link could exist between rhythm perception and the semantic content of words. The presentation of two syllable-timed languages and two mora-timed languages allows us to explore the possibility of a facilitation in sound symbolic relationship between mother tongue of participants and foreign languages. In this frame, we should expect a better performance for Finnish and Swahili than for Japanese and Tamil, in both the Italian and the Polish sample.

Moreover, while most of previous studies have employed as stimulus antonym pairs and their related translation in a forced choice task with two response alternatives, in this study we presented an auditory stimulus and three response options, thus reducing the possibility to guess the correct meaning by chance (from 50% with antonyms pairs to 33% with 3 response options).

Furthermore, to examine the contribution of sound symbolism in a cross-linguistic approach, we performed the experiment with native Italian and native Polish speakers, presenting to both of them the same unfamiliar languages. Thus, if a sound symbolic relation exists, we expect to find similar results in the two groups of participants as we hypothesize that the relation between words and meaning should not depend on participant’s mother tongue.

## Materials and Methods

### Participants

A total of 215 volunteers took part in the study: 149 participants were native Italian and lived in Italy at the time of the experiment (67 females; mean age = 26.62, standard error (SE) = 0.91), 66 were native Polish and lived in Poland at the time of the experiment (41 females; mean age = 20.85, SE = 0.91). None of the participants reported familiarity with the Finnish, Japanese, Tamil and Swahili languages and none of them complained hearing or speech impairments. Informed consent was obtained from all participants. The whole procedure was carried out in accordance with the principles of the Declaration of Helsinki, the protocol was approved by the Biomedical Research Ethics Committee, University of Chieti-Pescara, and participants gave written and informed consent before beginning the experiment.

### Stimuli and Procedure

The stimuli consisted of words selected primarily from the basic vocabulary of the Italian language. The words were therefore translated in the following languages: Finnish, Japanese, Swahili and Tamil. For each language a different pattern of 10 adjectives, 10 nouns and 10 verbs were presented, for a total of 120 stimuli (30 words for each foreign language, see Appendix). Audio files were generated using Google Translate voice, using the pronunciation in the target language (e.g., Japanese pronunciation for Japanese words), and were edited using GoldWave software (V.5.25; GoldWave Inc., Canada).

On each trial, the target word was presented acoustically via headphones and was followed by 3 response options presented in visual form (i.e., written on the computer monitor). One of the options was the correct translation of the target word, while the others were two distractors. The 3 response options were expressed in the mother tongue of participants and were chosen by individuals unaware of the aim of the study, checking that no phonological resemblance to the target word, nor a facilitation for the correct answer were present. For the Polish version of the experiment, the three response options were translated starting from the Italian ones, by a native Polish speaker with an excellent knowledge of the Italian language, who also excluded any phonological similarities among target word and response options.

Furthermore, in order to avoid a potential bias of the words length, both in the Italian and in the Polish version, the mean number of letters of the correct options was compared to the mean number of letters of the two distractors by means of *t*-tests. Results confirmed the absence of significant differences in length between correct and incorrect response options, for both Italian (*t*_119_ = -0.35, *p* = 0.723) and Polish words (*t*_119_ = -0.01, *p* = 0.988).

The 3 written options were arranged vertically on the monitor and were centered on the horizontal plane. Their vertical placement was balanced among trials so that the correct answer was presented either in the upper, central or lower position in an equal number of trials. The structure of the experiment was thus identical for the Italian and the Polish participants, with the only exception of the response options, which were presented in Italian or in Polish according to the nationality of the participant.

Participants were told that they would hear a word through the headphones in an unknown language followed by three translation options of that word written on the computer screen. They were instructed to try to select the word representing the more correct translation of the word they heard. They were asked to make their choice rapidly by clicking with the mouse on the word. For every trial the mouse cursor was reset in a central position.

The presentation order of auditory stimuli in the 4 languages was randomized. The total duration of the experiment was approximately 20 min. The experiment was run using a software written in E-Prime (Psychology Software Tools Inc., Pittsburgh, PA, United States) on a computer with a 15.4-inch monitor. The type of response (correct, incorrect) was automatically stored for subsequent analyses. Participants were tested in a quiet room; they sat comfortably in front of the computer monitor (approximately 70 cm from subject’s head) and wore a pair of headphones.

## Results

**FIGURE 1:**
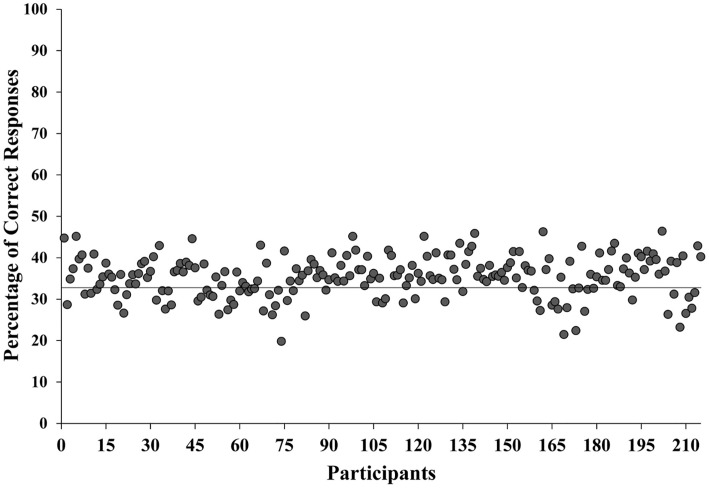
Mean percentage of correct responses for each participant. The horizontal line indicates the percentage due to the chance level (33.33%).

The number of correctly recognized words was transformed into percentages. Trials with a mean correct recognition above the 98.5 percentile and below the 1.5 percentile from the mean percentage were excluded from the analysis. This criterion was applied separately for the Italian and the Polish sub-samples, in order to exclude the possibility of phonetic similarity between the foreign languages presented and the mother tongue of participants. Thus, 5 words were excluded for each group of participants (2 Finnish, 2 Tamil and 1 Swahili for the Italian sample, and 2 Finnish and 3 Japanese for the Polish sample). The resulting mean accuracy of the whole sample was 35.31% which we compared with the threshold of the chance level by means of a single sample *t*-test against 33.33%, as 3 response options were presented for each stimulus. The difference was significant (*t*_214_ = 5.85, *p* < 0.001, see Figure 1), confirming that the correct recognition rate was higher than expected by chance.

A similar analysis was carried out for each of the 2 sub-samples, for each of the 4 foreign languages, as well as for each of the 3 categories of words. After the Bonferroni correction for multiple comparisons, the significant threshold was set at *p* = 0.025 for the 2 subsamples, *p* = 0.0125 for the 4 foreign languages, and it was set at *p* = 0.017 for the three categories of words. Results were significant for both the Italian (*t*_148_ = 5.16, *p* < 0.001) and the Polish subsample (*t*_65_ = 2.9, *p* = 0.005). Moreover, a *t*-test for independent groups showed that there was no difference in the performance between the 2 subgroups (*t*_213_ = -0.06, *p* = 0.949), thus all participants were considered together. Concerning the 4 languages, results were significant for Finnish (*t*_214_ = 6.13, *p* < 0.001) and for Japanese (*t*_214_ = 3.15, *p* = 0.002), whereas the recognition rate was not significantly better than chance for Swahili (*t*_214_ = 1.43, *p* = 0.155), and for Tamil (*t*_214_ = 1.63, *p* = 0.104), even if all of the recognition rates were higher than 33.33%. Considering the different categories of stimuli, verbs and nouns differed significantly from chance (verbs: *t*_214_ = 5.72, *p* < 0.001; nouns: *t*_214_ = 5.22, *p* < 0.001), whereas adjectives did not (*t*_214_ = -0.48, *p* = 0.634). Means, standard errors and *p*-values for all of these comparisons are reported in Table 1.

**Table 1:** Mean accuracy (percentage) and standard error (SE) and *p-*value.

	Mean	SE	*p-*value
Total	35.31	0.34	0.000*
Finnish	37.48	0.68	0.000*
Japanese	35.39	0.66	0.002*
Swahili	34.20	0.61	0.155
Tamil	34.22	0.56	0.104
Verbs	36.63	0.58	0.000*
Nouns	36.28	0.57	0.000*
Adjectives	33.09	0.50	0.634
Finnish-V	39.09	1.13	0.000*
Japanese-V	32.38	1.04	0.365
Swahili-V	37.91	1.11	0.000*
Tamil-V	36.93	1.03	0.001*
Finnish-N	41.09	1.20	0.000*
Japanese-N	37.26	1.13	0.001*
Swahili-N	32.75	1.03	0.575
Tamil-N	34.15	0.99	0.408
Finnish-A	32.53	0.96	0.409
Japanese-A	36.23	1.11	0.009*
Swahili-A	31.91	0.96	0.139
Tamil-A	31.72	0.89	0.071


*Data are reported as a total score and separately for languages (Finnish, Japanese, Swahili, Tamil), for categories (Verbs, Noun, Adjectives) and within each language by category. Asterisks show the significant results.*

In order to investigate more deeply possible differences among the four languages and the three categories of stimuli, taking into account the variability of subjects and items, we computed a mixed-model analysis of variance (ANOVA), using the variance estimation and precision module (VEPAC) of the Statistica 8 software (StatSoft Inc., Tulsa, 1984–2007). Post-hoc comparisons were carried out by means of LSD test and were corrected for multiple comparisons using Bonferroni correction.

In a first mixed-model ANOVA, Subject was included as a random factor, the percentage of correct recognitions was taken as the dependent variable, Group (Italian, Polish), Language (Finnish, Japanese, Swahili, Tamil) and Category (Noun, Verb, Adjective) were added to the model as fixed factors. The random effect Subject was significant (*F*_213,2343_ = 1.276 *p* = 0.006), whereas the fixed factor Group was not significant and it did not interact with the other factors. The fixed factor Language was significant (*F*_3,2343_ = 6.873, *p* < 0.001). Post-hoc comparisons revealed that the performance was higher for Finnish than for the other languages (Swahili and Tamil: *p* < 0.001). The fixed factor Category was significant (*F*_2,2343_ = 13.846, *p* < 0.001), and post-hoc comparisons showed that the performance was lower for adjectives with respect to both verbs and nouns (*p* < 0.001 for both comparisons). Finally, the interaction between the fixed factors Language and Category was significant (*F*_6,2343_ = 8.918, *p* < 0.001). For Finnish, the accuracy was lower with adjectives than with both verbs and nouns (*p* < 0.001 for both comparisons); for Tamil verbs were better recognized than adjectives (*p* = 0.013); for Swahili the performance was better with verbs than with both nouns (*p* = 0.002) and adjectives (*p* < 0.001); for Japanese it was lower with verbs than with nouns (*p* = 0.002). Moreover, nouns were better recognized in Finnish than in Swahili and Tamil (*p* < 0.001 for both comparisons), they were better recognized in Japanese than in Swahili (*p* = 0.003). Concerning verbs, the accuracy was lower for Japanese than for the other languages (Finnish: *p* < 0.001, Swahili: *p* = 0.001, Tamil: *p* = 0.023).

In a second mixed-model ANOVA, Item was included as random factor, percentage of correct recognitions was taken as the dependent variable, Group (Italian, Polish), Language (Finnish, Japanese, Swahili, Tamil) and Category (Noun, Verb, Adjective) were added to the model as fixed factors. The random effect Item was not significant, as well as the fixed factors Group and Language. The factor Group did not interact with the other factors. The fixed factor Category was significant (*F*_2,206_ = 5.108, *p* = 0.007), and post-hoc comparisons showed that the performance was lower for adjectives with respect to both verbs (*p* = 0.014) and nouns (*p* = 0.033). Finally, the interaction between the fixed factors Language and Category was significant (*F*_6,206_ = 3.103, *p* = 0.006). For Finnish, the accuracy was lower with adjectives than with nouns (*p* = 0.014). Moreover, nouns were better recognized in Finnish than in Swahili (*p* = 0.009).

## Discussion

The present study focused on the relationship between linguistic sound and meaning in the processing of real words. Contrary to what has been claimed for a long time in the linguistic domain about arbitrariness of language, our aim was to demonstrate that the sound of a word might contain a subtle connection with its meaning. Results of the present study provide support to this idea: indeed, overall we found that listeners reliably linked the acoustic properties of unfamiliar spoken words to the exact meaning at an above-chance level. This allows us to hypothesize that participants are sensitive to sound symbolic features of words and use them to guess the meaning of unknown words. It is noteworthy to remember that none of the participants reported exposure to the languages presented in this study, thus the sensitivity to these correspondences cannot be attributed to the listeners’ previous knowledge. Importantly, as hypothesized, the performance of Italian and Polish participants was not different, suggesting that the link between sound and meaning is an automatic and implicit process, possibly independent from the mother tongue of the listener. Furthermore, we observed a relatively high variability among subjects, not a surprising result considering the high number of participants and their inter-individual differences. The absence of differences between Italian and Polish participants (and the absence of interactions between Group and the other factors) supports the idea of a cross-linguistic generalizability of the sound symbolism effect, differently from the processing of a second language (D’Anselmo et al., 2013). These results are consistent with previous research (e.g., Yoshida, 2012) and suggest the existence of universal phonetic symbolism, which would be the basis of the recognition of correct meaning. This evidence is also supported by the mixed-model analysis by item, in which the random effect Item was not significant, confirming that regardless of the word used, in the present data the phonetic symbolism was a phenomenon cutting across the different languages. A very recent study demonstrated the existence of a sound symbolic relation among languages (Blasi et al., 2016). Performing a statistical examination of 100 words from 4298 different languages, the authors found that terms of unrelated languages contain the same sounds for specific referents (for example to indicate “smallness” concepts different languages include the high-front vowel /i/).

The original idea to investigate sound symbolism in the domain of natural languages using foreign (unknown) words has now achieved a rather long track of investigations (Brown et al., 1955; Maltzman et al., 1956; Brackbill and Little, 1957; Gebels, 1969; Imai et al., 2008). In one of these (Brown et al., 1955), participants were required to match antonymic pairs of foreign words, representing basic sensory parameters such as brightness/darkness or sharpness/bluntness, to their English equivalents. The study by Brown et al. (1955) demonstrated that sound symbolic patterns are evident even among sensory adjectives. However, certain changes in the experimental procedure, may lead to changes in performance. In the study of Maltzman et al. (1956) for example it was reproduced the experiment of Brown et al. (1955), with the variation that both stimulus and response options could be presented in languages unknown to participants. The error rate was higher when both stimulus and response were unknown. Brackbill and Little (1957) using a list of words not restricted to contrasting pairs (antonymic concepts), presented to subjects two words (from different languages) and asked to indicate if they had the same or different meaning. The performance was above chance but it was worse than in the original study of Brown et al. (1955).

In order to extend previous findings, in our study we have not presented strictly sensory words, furthermore we used a paradigm differing from those used before. Although previous studies have mostly used two-alternative forced choice paradigms, in which participants were asked to choose between two antonyms to guess the meaning of a spoken word (Tsuru and Fries, 1933; Brown et al., 1955), we proposed our participants a task with three response options, assuming that a larger set of alternatives would make the task more subtly discriminative, but not as demanding and indirect as with more response options (e.g., Aveyard, 2012). Probably with only two choices, the use of sound symbolic strategies is more immediate, it is possible that antonymic pairs exhibit a phonetic contrast that reflects their semantic contrast. Otherwise, with more response options participants are less aware about task manipulation and therefore less sensitive to the strategy useful to discriminate the meaning of words. Furthermore, in this study we presented linguistically unrelated response options and not antonyms as in previous studies, so that indirect facilitation effects should be limited. Under these assumptions, the results found in the present work are more likely to be strictly driven by a sound-meaning link, independently of conscious strategies.

If we look at the results for the different languages separately, we find that the sound symbolic effect is not significant with all of them; participants responded significantly better when words were presented in Finnish or Japanese, while responses to Swahili and Tamil stimuli did not reach significance. To exclude that these differences are due to a greater resemblance of some of these languages with Italian or with Polish, we purposely chose languages not belonging to the Indo-European family. These results, however, could be interpreted in the light of intrinsic differences among languages. For example some languages, such as Balto-Finnish or Japanese, seem to possess a rich ideophonic vocabulary (Hamano, 1998; Mikone, 2001) and this is consistent with our results regarding Finnish and Japanese. More specifically, we found that the performance in Finnish is better than that in all the other languages. As regards this last result it can not be excluded that, despite the different linguistic family, Germanic languages (belonging to the Indo-European family, as well as Italian and Polish) have somehow influenced the Finnish lexicon, since their geographical proximity. This speculation seems to support our results, but further studies are needed in order to disentangle this hypothesis.

It has to be noticed, however, that for all of the languages presented the performance was higher than chance, even if this difference was not always significant. Thus, the present results suggest that a tendency exists toward phonetic symbolism, and that it is observable in several languages, although with different intensities.

In this regard, the choice to present 3 response alternatives may have implied a limitation to significant results in all comparisons, but the tendency toward a recognition accuracy higher than chance in all languages should be anyway highlighted, all the more so since the word sounds were recorded using the pronunciation feature of Google Translate. It will be desirable for future studies to use stimuli recorded from natural voices since prosodic cues may convey referential information useful to infer word meaning (Tzeng et al., 2018), this would determine a more suitable comparison between languages, in terms of words rhythm perception. In this frame it has to be considered that we presented two syllable-timed languages (Finnish and Swahili) and two mora-timed languages (Japanese and Tamil), expecting to find a better performance for syllable-timed languages, due to the mother tongue of the participants tested (Italian and Polish). The results did not confirm this expectation, revealing a performance higher than chance level for Finnish and Japanese, suggesting that sound symbolism could be independent of the rhythmic structures of foreign languages.

It is possible that the difference among languages in the present study may be due to how they differ in their degree of iconicity. A probable origin of sound symbolism process can be explained assuming the existence of some kind of primitive imitative linkage between sound and meaning and this original iconicity could have had a role in the evolution of human language. As postulated by Brown and Nuttall (1959) the intersensory connections between language and meaning would be developed from language specific experience, rather than innately determined. Consequently, sound symbolism can clarify interesting aspects on how humans process languages. Furthermore, the sound symbolism that we investigated is not just a purely imitative process, such as in ideophones and in particular in onomatopoeias, in which a clear resemblance between sound and meaning is observed and a word represents the direct imitation of a sound. The sound symbolism investigated in this study instead is a more indirect process, that can be observed, in general words, and represents a more integrated feature of languages, probably resulting from a long period of language evolution. In other words, differently from onomatopoeias, in which the sound of the word represents the event which is described by the word, sound symbolism does not contain a direct link between the sound of the pronounced word and its meaning. This difference between symbolism and onomatopoeias leads to a crucial difference between the two: on one hand onomatopoeias are explicitly linked to their meaning and they are often shared explicitly by different languages, on the other hand sound symbolism is indirect and implicit, so that the possible link among different languages are not explicit and listeners can “guess” the correct meaning of a foreign word without the explicit knowledge of the word meaning.

The sensitivity of language users to iconic form meaning has been shown also in the domain of language learning. If on one hand it is true that arbitrariness facilitates the learning of specific word meanings (Monaghan et al., 2014) allowing for efficient communication, on the other hand some aspects of iconic form meaning represent a link between linguistic form and experience. Recent reviews support the notion that arbitrariness and iconicity coexist in language, each bringing its own benefits (Perniss et al., 2010; Perniss and Vigliocco, 2014). An interesting possibility is that sound symbolism may play a role in learning new words. A series of studies in this domain examined how children use this cross-modal association during early language development. Imai et al. (2008) found that Japanese children are facilitated in learning novel ideophonic verbs. This result was replicated in a study of Kantartzis et al. (2011) using the same sound symbolic verbs with English children, and providing evidence of a cross-linguistic early sensitivity in sound symbolism. A facilitation effect has been found also in a learning experiment with adults, in which sound symbolism enhanced learning of Japanese ideophones when they were matched with the real Dutch translations, but not when they were matched with their opposite Dutch translations (Lockwood et al., 2016).

Recent investigations have supported sound symbolism theories from a neuroimaging perspective, providing interesting suggestions about the comprehension of how the brain perceives, processes and constructs sound symbolism. Basically, these studies showed that sound symbolic words are processed differently from other words in the human brain, with a greater engagement of cross-modal sensory integration processes (Revill et al., 2014). In a fMRI investigation, Kanero et al. (2014) showed that Japanese ideophones elicited more activity in the right superior temporal sulcus (STS), differently from non sound symbolic verbs and adverbs. They hypothesize that the posterior STS plays a critical role in sound symbolism processing probably working as a center of multimodal integration useful for the processing of iconic and arbitrary aspects. Furthermore, Lockwood and Tuomainen (2015) in a EEG study found that ideophones compared to arbitrary words, elicited a greater P2 and they argue that this late positive complex is distinctive of ideophone processing, which engages an integration of sound and sensory aspects (for a review see Lockwood and Dingemanse, 2015).

Neuroimaging studies in this domain are not numerous, therefore further investigation should be performed focusing on the brain processing of words in multiple unknown foreign languages. Given the evidence of phonetic sound symbolism also at a neuronal level, it can be assumed that a correlation exists among words better recognized, which are probably more sound symbolic and the involvement of areas of cross-modal integration.

## Conclusion

In conclusion the series of research in the domain of sound symbolism reveals that this is not a marginal aspect in linguistics. In this study we found that participants perform better than chance in the recognition of unknown words in natural languages and we attribute this results to a sound symbolic component of languages. Further studies are needed to explore sound symbolism, also comparing different languages, since it could shed new light on important questions about the origin and evolution of languages and can help to better understand how linguistic and psychological mechanisms interact in the perception and understanding of words.

## Author Contributions

AD and AB designed the paradigm. AD and PZ collected the data. AD and GP analyzed and interpreted the data and wrote the draft. AB and LT critically revised the manuscript.

## Conflict of Interest Statement

The authors declare that the research was conducted in the absence of any commercial or financial relationships that could be construed as a potential conflict of interest.

## Appendix

**Table A1:** List of stimulus words.

Category	Language	Phonetic	Correct Italian	Option 1	Option 2	Correct Polish	Option 1	Option 2
**VERBS**								
	Finnish	*hyppiä*	**saltare**	amare	uccidere	**skoczyć**	kochać	zabić
	Finnish	*terve*	**salutare**	indossare	salire	**przywitać się**	nosić	wspinać się
	Finnish	*avoin*	**aprire**	sognare	lasciare	**otworzyć**	marzyć	odejść
	Finnish	*märkä*	**bagnare**	puntare	finire	**zmoczyć**	wbijać	zakończyć
	Finnish	*kiitos*	**ringraziare**	onorare	combattere	**dziękować**	zaszczycać	walczyć
	Finnish	*lakaista*	**spazzare**	correre	spostare	**zamiatać**	biegać	przesunąć
	Finnish	*kampa*	**pettinare**	partorire	accoppiare	**czesać**	rodzić	do pary
	Finnish	*osoittaa*	**indicare**	fotografare	captare	**wskazać**	fotografować	złapać
	Finnish	*ratsastaa*	**cavalcare**	contare	bere	**jeździć konno**	liczyć	pić
	Finnish	*rauta*	**stirare**	diventare	attraversare	**prasować**	stać się	przejść przez
	Japanese	*kaku*	**scrivere**	fotografare	pensare	**pisać**	fotografować	myśleć
	Japanese	*teishutsu suru*	**sottomettere**	sistemare	contare	**podporządkować**	układać	liczyć
	Japanese	*pairotto*	**pilotare**	insegnare	colpire	**pilotować**	trafić	uczyć
	Japanese	*hikari*	**illuminare**	cantare	nascere	**rozświetlić**	śpiewać	urodzić się
	Japanese	*seichō suru*	**crescere**	sparare	contare	**rosnąć**	strzelać	liczyć
	Japanese	*korosu*	**uccidere**	mangiare	aprire	**zabić**	jeść	otworzyć
	Japanese	*iku*	**andare**	nuotare	leggere	**iść**	pływać	czytać
	Japanese	*egaku*	**disegnare**	correre	accendere	**rysować**	biegać	włączyć
	Japanese	*jōshō*	**salire**	comandare	capire	**wspinać się**	dowodzić	zrozumieć
	Japanese	*nioi o kagu*	**annusare**	toccare	spostare	**wąchać**	dotknąć	przesunąć
	Swahili	*rasha*	**imbiancare**	pilotare	colpire	**wybielać**	pilotować	trafić
	Swahili	*kuondoa*	**rimuovere**	girare	falciare	**usunąć**	obracać	kosić
	Swahili	*urekebishaji*	**ristrutturare**	vivere	stirare	**zrestrukturyzować**	mieszkać	prasować
	Swahili	*kuangalia*	**controllare**	aprire	uccidere	**kontrolować**	otwierać	zabić
	Swahili	*rudia*	**rifare**	sparare	liberare	**przerobić**	strzelać	wypuścić
	Swahili	*kusamehe*	**perdonare**	formulare	soccombere	**przebaczyć**	formułować	ulec
	Swahili	*kustawi*	**fiorire**	proporre	vivere	**zakwitnąć**	zaproponować	żyć
	Swahili	*kuiga*	**imitare**	volare	telecomandare	**imitować**	latać	zdalnie sterować
	Swahili	*kutamani*	**ambire**	fantasticare	nuotare	**aspirować**	marzyć	pływać
	Swahili	*kujenga*	**costruire**	contare	nascondere	**budować**	liczyć	ukryć
	Tamil	*aṭaiya*	**raggiungere**	volare	capire	**dotrzeć**	latać	rozumieć
	Tamil	*piu vāṅku*	**ripercorrere**	piangere	soccombere	**przebyć znowu**	płakać	ulec
	Tamil	*āu*	**accendere**	restare	odiare	**włączyć**	zostać	nienawidzić
	Tamil	*kik*	**calciare**	inventare	piacere	**kopnąć**	wymyślać	lubić
	Tamil	*erukṭalalakai*	**affascinare**	giocare	starnutire	**fascynować**	grać	kichać
	Tamil	*taṇṭikka*	**punire**	inviatare	scavare	**ukarać**	zapraszać	kopać
	Tamil	*eṉṟu*	**pensare**	istruire	arrivare	**myśleć**	instruować	przybyć
	Tamil	*caṇṭai*	**combattere**	risalire	annusare	**walczyć**	wspiąć się ponownie	wąchać
	Tamil	*mukam*	**affrontare**	comandare	capire	**stawić czoła**	dowodzić	zrozumieć
	Tamil	*uyartta*	**alzare**	provare	dirigere	**podnieść**	próbować	kierować
**NOUNS**								
	Finnish	*päivä*	**giorno**	tavolo	suono	**dzień**	stół	dźwięk
	Finnish	*yö*	**notte**	pietra	starnuto	**noc**	kamień	kichnięcie
	Finnish	*aamu*	**mattina**	sbadiglio	tuffo	**poranek**	ziewnięcie	nurkowanie
	Finnish	*terveys*	**salute**	fiore	piatto	**zdrowie**	kwiat	kwiat
	Finnish	*vanhemmat*	**genitori**	sedia	muscolo	**rodzice**	krzesło	mięsień
	Finnish	*ystävä*	**amico**	ape	onda	**przyjaciel**	pszczoła	fala
	Finnish	*moraalinen*	**morale**	semaforo	segnalibro	**moralność**	sygnalizacja świetlna	zakładka
	Finnish	*nainen*	**donna**	onda	treccia	**kobieta**	fala	warkocz
	Finnish	*käännös*	**traduzione**	sale	partita	**tłumaczenie**	sól	mecz
	Finnish	*uni*	**sonno**	unghia	pomeriggio	**sen**	paznokieć	popołudnie
	Japanese	*Kasai*	**fuoco**	coltello	scrittura	**ogień**	nóż	pismo
	Japanese	*mizu*	**acqua**	compagno	viaggio	**woda**	kompan	podróż
	Japanese	*kawa*	**fiume**	musica	unghia	**rzeka**	muzyka	paznokieć
	Japanese	*umi*	**mare**	paura	pianto	**morze**	strach	płacz
	Japanese	*purinta*	**stampante**	cattedra	cucina	**drukarka**	katedra	kuchnia
	Japanese	*kodomo*	**bambino**	rosso	tempo	**dziecko**	czerwone	czas
	Japanese	*skari*	**ragazzo**	qualità	parametro	**chłopiec**	jakość	parametr
	Japanese	*hōmu*	**casa**	corsa	stomaco	**dom**	wyścig	żołądek
	Japanese	*rekōdā*	**registratore**	pulsante	vasca	**rejestrator**	przycisk	wanna
	Japanese	*dōbutsu*	**animale**	mucca	guancia	**zwierzę**	krowa	policzek
	Swahili	*wingu*	**nuvola**	tuono	crescita	**chmura**	grom	wzrost
	Swahili	*hamu*	**desiderio**	suono	cuore	**pożądanie**	dźwięk	serce
	Swahili	*kifo*	**morte**	inganno	cerchio	**śmierć**	oszustwo	okrąg
	Swahili	*chakula*	**cibo**	peso	velocità	**jedzenie**	waga	prędkość
	Swahili	*samaki*	**pesce**	vagabondo	età	**ryba**	włóczęga	wiek
	Swahili	*ardhi*	**terra**	comportamento	ombra	**ziemia**	zachowanie	cień
	Swahili	*maisha*	**vita**	testa	bianco	**życie**	głowa	biały
	Swahili	*chaki*	**gesso**	zaino	chiavi	**gips**	plecak	klucze
	Swahili	*jiwe*	**pietra**	tensione	difficoltà	**kamień**	napięcie	trudność
	Swahili	*matunda*	**frutto**	pesca	errore	**owoc**	wędkarstwo	pomyłka
	Tamil	*cûriyaṉ*	**sole**	nonno	racconto	**słońce**	dziadek	historia
	Tamil	*kāl*	**gamba**	mare	singhiozzo	**noga**	morze	łkanie
	Tamil	*cantiraṉ*	**luna**	mela	carta	**księżyc**	jabłko	papier
	Tamil	*tolaipçci*	**telefono**	macchina	maniglia	**telefon**	maszyna	uchwyt
	Tamil	*mārpu*	**seno**	parola	dolore	**pierś**	słowo	ból
	Tamil	*nābrkāli*	**sedia**	muscolo	maglione	**krzesło**	mięsień	sweter
	Tamil	*naṭcattira*	**stella**	energia	bestiame	**gwiazda**	energia	żywy inwentarz
	Tamil	*eṟumpu*	**formica**	fiore	pesca	**mrówka**	kwiat	brzoskwinia
	Tamil	*ākāyam*	**cielo**	vita	conoscenza	**niebo**	życie	wiedza
	Tamil	*çṟu*	**salita**	discesa	altura	**wspinaczka**	zjazd	wyżyna
**ADJECTIVES**								
	Finnish	*viisas*	**saggio**	vile	coraggioso	**mędrzec**	tchórzliwy	odważny
	Finnish	*hereillä*	**sveglio**	amaro	sconnesso	**obudzony**	gorzki	odłączony
	Finnish	*vaikea*	**severo**	divertente	saggio	**surowy**	zabawny	mędrzec
	Finnish	*julma*	**crudele**	silenzioso	magico	**okrutny**	milczący	magiczny
	Finnish	*hauska*	**divertente**	elegante	**zabawny**	elegancki	śpiący
	Finnish	*kaunis*	**bello**	maschile	preoccupato	**piękny**	męski	zmartwiony
	Finnish	*alhainen*	**basso**	semplice	vicino	**niski**	prosty	blisko
	Finnish	*epäpyhä*	**profano**	ordinato	**bluźnierczy**	zamówić	nieprzewidywalny
	Finnish	*rohkea*	**coraggioso**	estremo	muscoloso	**odważny**	ekstremalny	muskularny
	Finnish	*pikkulasten*	**infantile**	laborioso	memorabile	**dziecinny**	pracochłonny	niezapomniany
	Japanese	*mujakina*	**innocente**	ingiusto	crudo	**niewinny**	niesprawiedliwy	surowy
	Japanese	*taikutsuna*	**noioso**	dolce	colorato	**nudny**	słodki	kolorowy
	Japanese	*ō*	**ampio**	colorato	cattivo	**szeroki**	kolorowy	zły
	Japanese	*nagai*	**lungo**	melodico	intelligente	**długi**	melodyczny	inteligentny
	Japanese	*hageshî*	**violento**	elegante	infinito	**przemocowy**	elegancki	nieskończony
	Japanese	*ningen*	**umano**	fragile	animato	**ludzki**	kruchy	animowany
	Japanese	*passhibu*	**passivo**	ricco	fastidioso	**pasywny**	bogaty	przykry
	Japanese	*furu*	**pieno**	vuoto	intrepido	**pełny**	pusty	nieustraszony
	Japanese	*takai*	**alto**	tenero	piacevole	**wysoki**	czuły	przyjemny
	Japanese	*mugen*	**infinito**	scaltro	acceso	**nieskończony**	przebiegły	zapalony
	Swahili	*rangi*	**colorato**	familiare	sbagliato	**kolorowy**	członek rodziny	niewłaściwy
	Swahili	*mungu*	**divino**	paziente	pesante	**boski**	cierpliwy	ciężki
	Swahili	*hila*	**astuto**	ingenuo	universale	**przebiegły**	naiwny	uniwersalny
	Swahili	*ngumu*	**difficile**	interrotto	velenoso	**trudny**	przerwany	trujący
	Swahili	*kelele*	**rumoroso**	socievole	severo	**hałaśliwy**	towarzyski	surowy
	Swahili	*mafuta*	**grasso**	fumoso	fresco	**gruby**	dymiący	świeży
	Swahili	*giza*	**buio**	morbido	aspro	**ciemny**	miękki	kwaśny
	Swahili	*amelala*	**addormentato**	colpevole	profano	**śpiący**	winny	sprofanowany
	Swahili	*hasa*	**particolare**	immobile	solitario	**szczególny**	nieruchomy	odosobniony
	Swahili	*baridi*	**freddo**	morbido	rotondo	**zimny**	miękki	okrągły
	Tamil	*nōyāḷi*	**paziente**	buio	cotto	**cierpliwy**	ciemny	gotowany
	Tamil	*icai cārnta*	**melodico**	commestibile	terrificante	**melodyczny**	jadalny	przerażający
	Tamil	*amaitiyāka*	**silenzioso**	stupido	protetto	**cichy**	głupi	zabezpieczony
	Tamil	*irakaciyamāka*	**segreto**	silenzioso	contagioso	**sekretny**	cichy	zaraźliwy
	Tamil	*vçkamāka*	**veloce**	finito	grande	**szybki**	skończony	duży
	Tamil	*māṟāka*	**contrario**	naturale	creativo	**sprzeczny**	naturalny	kreatywny
	Tamil	*kuṭumpa*	**familiare**	profondo	codardo	**rodzinny**	głęboki	tchórz
	Tamil	*cikkalāṉa*	**complesso**	semplice	goloso	**kompleksowy**	prosty	łakomy
	Tamil	*veḷi*	**esterno**	segreto	vile	**zewnętrzny**	sekretny	tchórzliwy
	Tamil	*poyyaṉ*	**bugiardo**	esile	vero	**kłamliwy**	cienki	prawdziwy

*First column: word category (verbs, nouns, and adjectives); second column: foreign Language (Finnish, Japanese, Swahili, and Tamil); third column: phonetic transcription of the sound of the word. The columns “Correct Italian” and “Correct Polish” indicate, respectively, the correct Italian and Polish translation of the auditory word presented. The columns “Option 1” and “Option 2” indicate the two incorrect response alternatives in both Italian and Polish.*
